# Runx-dependent and silencer-independent repression of a maturation enhancer in the *Cd4* gene

**DOI:** 10.1038/s41467-018-05803-3

**Published:** 2018-09-05

**Authors:** Satoshi Kojo, Nighat Yasmin, Sawako Muroi, Mari Tenno, Ichiro Taniuchi

**Affiliations:** 1 Laboratory for Transcriptional Regulation, RIKEN Center for Integrative Medical Sciences (IMS), 1-7-22 Suehiro-cho, Tsurumi-ku, Yokohama 230-0045 Japan; 2 Present Address: Department of Biomedical Sciences, King Edward Medical University Nelagumbad Mayo Hospital Road, Lahore, 54000 Pakistan

## Abstract

An intronic silencer, *S4*, in the *Cd4* gene has been shown to be responsible for the helper-lineage-specific expression of CD4; *S4* requires Runx complex binding to exert its silencer function against the enhancer-mediated *Cd4* activation by modulating the epigenetic state of the *Cd4* gene. Here we identify a late-acting maturation enhancer. Bcl11b plays essential roles for activation of both the early-acting proximal enhancer and maturation enhancer of *Cd4*. Notably, Runx complexes suppress these enhancers by distinct mechanisms. Whereas repression of the proximal enhancer depends on the *S4* silencer, the maturation enhancer is repressed by Runx in the absence of *S4*. Moreover, ThPOK, known to antagonize *S4*-mediated *Cd4* repression, assists Runx complexes to restrain maturation enhancer activation. Distinct modes of *S4* silencer action upon distinct enhancers thus unravel a pathway that restricts CD4 expression to helper-lineage cells by silencer-independent and Runx-dependent repression of maturation enhancer activity in cytotoxic-lineage cells.

## Abstract (web summary)

The commitment of helper and cytotoxic lineages for CD4 and CD8 T cells, respectively, is associated with the regulation of *Cd4* gene expression. Here the authors show that an intronic silencer, *S4*, has differential effects and synergy with the RUNX complex to act on two enhancer elements of the CD4 gene to control T cell lineage commitment in the thymus.

## Introduction

CD4 and CD8 glycoproteins function as a co-receptor that assists T-cell antigen receptor (TCR) to recognize antigenic peptide presented by major histocompatibility complex (MHC) class II and class I molecules, respectively^1^. In addition, CD4/CD8 molecules serve as useful markers to define thymocyte developmental stages and helper-lineage and cytotoxic-lineage T cells^2^. Signals from pre-TCR complexes in CD4^−^CD8^−^ double-negative (DN) thymocyte progenitors induce both CD4 and CD8 expression, resulting in the generation of CD4^+^CD8^+^ double-positive (DP) precursor thymocytes. A limited numbers of DP thymocytes, which have passed a process known as positive selection, differentiate further into mature thymocytes^3^. Post-selection thymocytes expressing MHC-class I (MHC-I) restricted TCRs are specified to differentiate into the cytotoxic-lineage and acquire CD4^−^CD8^+^ single-positive (SP) phenotype by terminating CD4 expression, whereas MHC-class II (MHC-II)-mediated TCR engagement generates CD4^+^CD8^−^ SP thymocytes committed to the helper-lineage by inhibiting CD8 expression.

Such stage-specific and lineage-specific expression of CD4/CD8 co-receptors is regulated at the transcriptional level by a combinational regulation of *cis*-regulatory elements^2^. For instance, insertion of a 434 bp intronic transcriptional silencer (*S4*) into a transgene together with a 430 bp proximal enhancer (*E4p*) and a minimal *Cd4* promoter (*P4)* is necessary to recapitulate stage-specific and lineage-specific *Cd4* expression in reporter transgene expression^4,5^. CD4 de-repression from CD8^+^ T cells upon ablation of the *S4* sequences^6,7^. These observations established a model that the single *S4* silencer controls helper-lineage specific expression of the *Cd4* gene^8^. Sequential studies further revealed that binding of Runx transcription factor complexes to *S4* through their recognition of two Runx-motifs is essential for *S4* activity^9,10^.

Ablation of the *E4p* from the murine *Cd4* locus (*Cd4*^*ΔE4p/ΔE4p*^ mice) also confirmed that *E4p* is essential to initiate *Cd4* activation^11^. However, despite severely diminished CD4 expression on precursor thymocytes, a small but significant proportion of precursors was positively selected and differentiated into mature thymocytes expressing CD4 at a lower level in *Cd4*^*ΔE4p/ΔE4p*^ mice, leading to an assumption that additional enhancer(s), referred to as a maturation enhancer (*E4m*), should be present^11^. It has also been shown that stage-specific DNA de-methylation in helper-lineage cells and continuous DNA methylation in cytotoxic-lineage cells are involved in establishment of the heritable active and silent status, and requires *E4p* and *S4* activity, respectively^11,12^. Thus, *Cd4* gene regulation has served as an ideal model to study how stage-specific and lineage-specific epigenetic modifications are regulated by *cis*-regulatory elements. However, the genomic region containing *E4m* activity remains elusive, as does the mechanism by which *E4m* activity is regulated.

In this study, we identify the *E4m*, validated its role in regulating CD4 expression, and isolate Bcl11b as an important activator for *E4m*. We also show that Runx complexes repress the *E4m* activity in CD8^+^ T cells even in the absence of the *S4* and find unexpected ThPOK function that prevents premature *E4m* activation by assisting Runx-mediated *E4m* repression. Collectively, our results reveal that Runx complexes repress two enhancers, *E4p* and *E4m*, in distinct manners, providing a novel insight that revises a silencer-based model for a lineage-specific *Cd4* expression.

## Results

### Rescue of *E4p* function by a heterologous enhancer

**Fig. 1:**
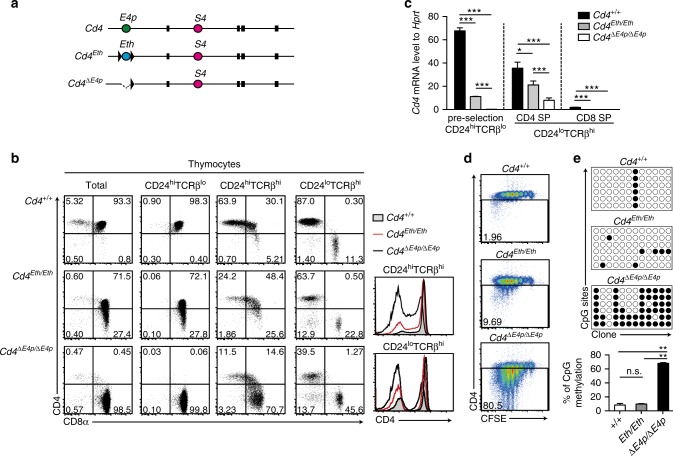
Enhancer replacement between *Cd4* and *Thpok* genes. **a** Schematic structures of mutant *Cd4* alleles. Ovals marked with different colors represent c*is*-regulatory regions, *Cd4* silencer (*S4*), *Cd4* proximal enhancer (*E4p*), and synthetic *Thpok* enhancer (*Eth*). The black box and triangle represent exons and loxP sequences, respectively. **b** Dot plots showing CD4 and CD8 expression and histograms at the right showing CD4 expression in indicated cell subsets from mice with indicated genotypes. Numbers in quadrants indicate respective cell percentage. Representative results of at least three independent analyses. **c** Graph showing relative *Cd4* to *Hprt* mRNA in pre-selection CD24^hi^TCRβ^lo^ thymocytes, CD24^lo^TCRβ^hi^ CD4 single positive (SP), and CD24^lo^TCRβ^hi^ CD8 SP thymocytes of mice with indicated genotypes. Means ± SD. ***p < 0.001, *p < 0.05 ((One-way ANOVA and Tukey’s multiple comparison test)). **d** Pseudocolor plots showing CD4 expression and cell divisions five days after in vitro stimulation of sorted CD4^+^ T cells from mice with indicated genotype. Numbers in the indicated gate represent cell percentages. **e** Bisulfite PCR at six amplicons from the first intron in the *Cd4* gene in naïve CD4^+^ T cells from mice with indicated genotypes. Symbols indicate methylated (black filled circle) or un-methylated (black open circle) CpG motifs. The lower graph shows the summary of three independent experiments. Means ± SD. ***p < 0.01 (unpaired student *t* test, two-sided)

It was shown that *E4p* is necessary for DNA de-methylation of the *Cd4* gene^12^. To examine whether the activity that induces DNA de-methylation in the *Cd4* locus is specific to *E4p*, we tested how a heterologous enhancer behaves in the *Cd4* locus. Two enhancers, a thymic enhancer (*TE*) and a proximal enhancer (*PE*), are present in the *Thpok* gene encoding the CD4-specific transcription factor ThPOK^13,14^. Low expression of *Thpok* upon removal of Tet family proteins that are essential for DNA de-methylation^15^ suggests an involvement of DNA de-methylation in activation of the *Thpok* gene. In order to replace *E4p* sequence in the *Cd4* locus with the two separately located enhancers in the *Thpok* locus, we synthesized an *Eth* DNA fragment in which core sequences of *TE* and *PE* were conjugated (Supplementary Fig. 1a), and generated a *Cd4*^*Eth*^ allele through homologous recombination in embryonic stem (ES) cells (Fig. 1a and Supplementary Fig. 1b).

CD4 expression on *Cd4*^*Eth/Eth*^ thymocytes at the DP stage, defined as the CD24^hi^TCRβ^lo^ population, was lower than that in control *Cd4*^*+/+*^ but higher than that in *Cd4*^*ΔE4p/ΔE4p*^ cells (Fig. 1b, c). Given that the activity of *TE* and *PE* in the *Thpok* locus was low in pre-selection DP thymocytes and gradually increased during thymocytes maturation^13,14,16^, *Eth* activity in the *Cd4* locus was likely to retain original stage-specificity and not be sufficient to completely restore CD4 expression in precursor DP thymocytes. However, during maturation into the helper-lineage, the CD4 expression level from the *Cd4*^*Eth*^ allele closely approached that from the *wild-type Cd4* allele (Fig. 1b, c, and Supplementary Fig. 1c).

We next examined whether CD4 expression from the *Cd4*^*Eth*^ allele could be stably maintained after cell division. Although the CD4 expression level from the *Cd4*^*ΔE4p*^ allele was decreased according to the rounds of cell divisions as previously reported^11,12^, CD4 expression was retained at a higher level in CD4^+^ cells from *Cd4*^*Eth/Eth*^ mice (Fig. 1d). DNA de-methylation at the intronic region was induced in *Cd4*^*Eth/Eth*^ cells to a similar extent as that in control *Cd4*^*+/+*^ cells, whereas DNA remained significantly methylated in *Cd4*^*ΔE4p/ΔE4p*^ cells (Fig. 1e). These observations indicated that the synthetic heterologous *Eth* enhancer was able to restore *E4p* function in the *Cd4 locus* to a degree sufficient to establish a heritable actives state at least in part through the induction of DNA de-methylation.

### Characterization of the *Cd4* maturation enhancer (*E4m*)

**Fig. 2:**
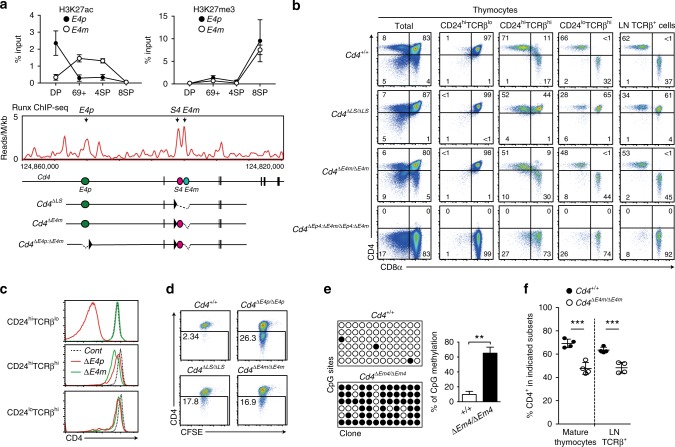
Effects of loss of the maturation enhancer, *E4m*, on CD4 expression. **a** Upper graphs showing kinetic changes of H3K27ac and H3K27me3 at *E4p* and *E4m* regions in pre-selection (DP), freshly selected (CD69^+^), and CD4 SP (4SP) and CD8 SP (8SP) thymocytes. Summary of three independent ChIP experiments. Means ± SD. (Middle) Runx ChIP-Seq track at the *Cd4* gene in total thymocytes. Gene structures and regulatory regions are shown as in Fig. 1a. Schematic structures of mutant *Cd4* alleles are shown at the bottom. **b** Pseudocolor plots showing CD4 and CD8 expression in indicated cell subsets from mice with indicated genotypes. Numbers in quadrants indicate respective cell percentages. Representative results of at least three independent analyses of each genotype. **c** Histograms showing CD4 expression levels on CD24^hi^TCRβ^lo^, CD8-negative CD24^hi^TCRβ^hi^ and CD8-negative CD24^lo^TCRβ^hi^ thymocytes from *Cd4*^*+/+*^ (dotted), *Cd4*^*ΔE4p/ΔE4p*^ (red), and *Cd4*^*ΔE4m/ΔE4m*^ (green) mice. One representative of three analyses. **d** Pseudocolor plots showing CD4 expression and cell divisions five days after in vitro stimulation of sorted peripheral CD4^+^ T cells from mice with indicated genotypes. Numbers in the indicated gate represent cell percentages. **e** Bisulfite PCR analyses of naïve CD4^+^ T cells from *Cd4*^*+/+*^ and *Cd4*^*ΔE4m/ΔE4m*^ mice are shown as in Fig. 1d. The right graph shows a summary of three independent experiments. Means ± SD. **p < 0.01 (unpaired student *t* test, two-sided). **f** Graph showing percentage of CD4^+^CD8^−^ cells in mature (CD24^lo^TCRβ^hi^) thymocytes and the lymph node TCRβ^+^ population of *Cd4*^*+/+*^ and *Cd4*^*ΔE4m/ΔE4m*^ mice. Means ± SD. ***p < 0.001 (unpaired student *t* test, two-sided)

In the murine *Cd4* locus, the presence of a *Cd4* maturation enhancer (*E4m*) shortly downstream of *S4* has been predicted^17^. We found conserved sequences at approximately 1 kb downstream of the *S4* region (Supplementary Fig. 2a); moreover, the public ATAC-Seq database showed that this region become more accessible specifically in CD4-lineage cells after positive selection, whereas the *S4* region became more accessible in CD8-lineage cells (Supplementary Fig. 2a). In addition, our chromatin immunoprecipitation (ChIP) combined with sequencing (ChIP-Seq) analysis detected binding of Runx complexes to *S4* and its downstream region in total thymocytes (Fig. 2a). Furthermore, changes of post-translational modifications on K27 of histone 3 for tri-methylation (H3K27me3) and for acetylation (H3K27ac), which are known as markers for inactive and active enhancer states^18^, respectively, indicated that H3K27ac at *E4p* was highest in precursor DP thymocytes, whereas H3K27ac was increased at a the putative *E4m* region in post-selection (CD69^+^CD24^hi^TCRβ^+^) thymocytes. In comparison, H3K27me3 modification accumulated at both regions specifically in CD8^+^ T cells. Together, these features prompted us to test the function of this putative *E4m* region genetically by deleting the 337 bp core conserved sequences. Accordingly, by using an ES cell clone harboring a *Cd4*^*+/+*^ or *Cd*^*ΔS4/ΔE4p*^ genotype (Supplementary Fig. 2b, c), we established two mouse lines harboring *Cd4*^*ΔE4m*^ and *Cd4*^*ΔE4p:ΔE4m*^ alleles, with the latter lacking both *E4p* and *E4m* (Supplementary Fig. 2b, c). Strikingly, CD4 expression during T cell development was completely abolished in the *Cd4*^*ΔE4p:ΔE4m/ΔE4p:ΔE4m*^ mice (Fig. 2b). This finding demonstrated not only that enhancer activity complementing CD4 expression (*E4m*) from the *Cd4*^*ΔE4p*^ allele is embedded on the deleted region, but also that *E4p* and *E4m* serve as major enhancers to drive CD4 expression in T cells.

We next examined the impact of the *E4m* deletion alone on CD4 expression and T cell development in *Cd4*^*ΔE4m/ΔE4m*^ mice. Although the CD4 expression level at the DP stage was compatible between *Cd4*^*+/+*^ and *Cd4*^*ΔE4m/ΔE4m*^ cells, it became lower in *Cd4*^*ΔE4m/ΔE4m*^ cells than that in *Cd4*^*+/+*^ cells at the CD8^−^CD24^hi^TCRβ^hi^ stage (Fig. 2b, c). Notably, CD4 expression level from the *Cd4*^*ΔE4p*^ allele was higher than that from the *Cd4*^*ΔE4m*^ allele in CD8^−^CD24^hi^TCRβ^hi^ cells, although CD4 expression levels from these two mutant *Cd4* alleles became similar to each other and lower than that from the wild-type *Cd4* allele in mature (CD24^lo^TCRβ^hi^) CD8^−^ thymocytes. Thus, the *Cd4*^*ΔE4p*^ allele could induce CD4 expression faster than the *Cd4*^*ΔE4m*^ allele after positive selection, although changes of histone modification patterns at the *E4p* was equivalent between control *Cd4* and *Cd4*^*ΔE4m*^ alleles (Supplementary Fig. 3).

Following activation of CD4^+^CD8^−^ splenic T cells, CD4 expression from the *Cd4*^*ΔE4m*^ allele decreased to a similar extent as that from the *Cd4*^*ΔLS*^ allele (Fig. 2d), in which both *S4* and *E4m* were deleted^6^. However, the percentage of cells retaining CD4 expression was higher in *Cd4*^*ΔE4m/ΔE4m*^ cells than in *Cd4*^*ΔE4p/ΔE4p*^ cells, although the percentage of methylated CpG motifs at intronic regions remained higher in these two cell types to a similar extent (Figs 1c and 2e). These results indicated that *E4m* activity is also necessary to establish a heritable active state at the *Cd4* locus in part by activating the DNA de-methylation process together with *E4p*.

**Fig. 3:**
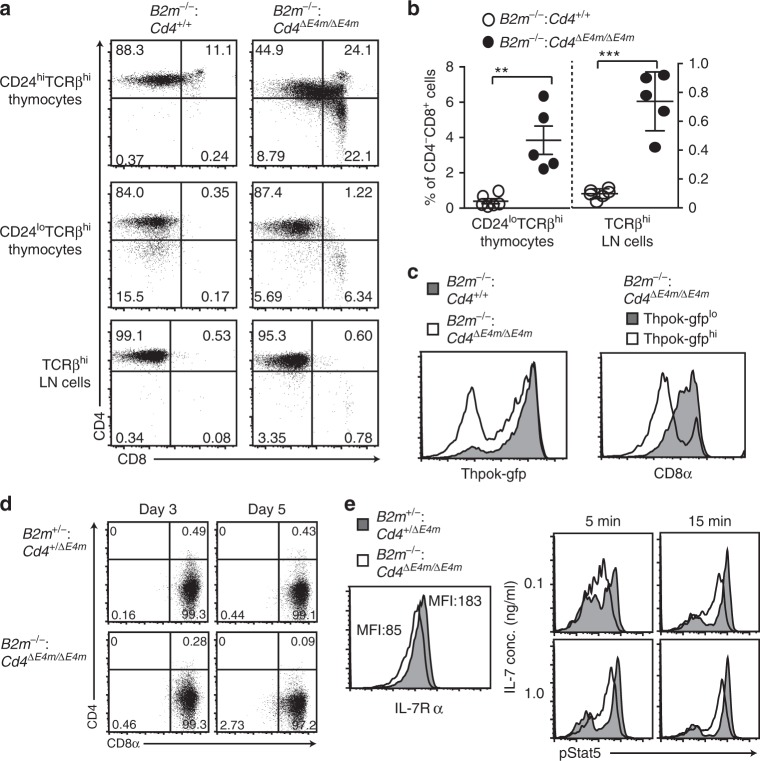
Re-directed differentiation of MHC-II selected thymocyte by loss of the *E4m* enhancer. **a**, **b** Dot plots in **a** showing CD4 and CD8 expression in indicated cell subsets from *B2m*^*−/−*^: *Cd4*^*+/+*^ and *B2m*^*−/−*^: *Cd4*^*ΔE4m/ΔE4m*^ mice. Graphs in **b** showing summary of percentage of CD4^−^CD8^+^ cells in mature (CD24^lo^TCRβ^hi^) thymocytes and the lymph node (LN) TCRβ^+^ population. Means ± SD. **p < 0.01, ***p < 0.001 (unpaired student *t* test, two-sided). **c** Histogram at left showing Thpok-gfp expression in freshly-selected (CD24^hi^TCRβ^hi^) thymocytes of *B2m*^*−/−*^: *Cd4*^*+/+*^ and *B2m*^*−/−*^: *Cd4*^*ΔE4m/ΔE4m*^ mice. Histogram at right showing CD8 expression in Thpok-gfp^lo^ and Thpok-gfp^hi^ CD24^hi^TCRβ^hi^ thymocytes of *B2m*^*−/−*^: *Cd4*^*ΔE4m/ΔE4m*^ mice. One representative result of two mice. **d** Dot plots showing CD4 and CD8 expression three and five days after TCR stimulation of sorted CD4^−^CD8^+^ mature thymocytes from *B2m*^*+/−*^: *Cd4*^*+/ΔE4m*^ and *B2m*^*−/−*^: *Cd4*^*ΔE4m/ΔE4m*^ mice. Numbers in quadrants indicate respective cell percentages. **e** IL7R expression in CD4^−^CD8^+^ thymocytes (left) and phosphorylated Stat5 levels in CD4^−^CD8^+^ thymocytes (right) at 5 and 15 min following IL7 stimulation at indicated concentrations from *B2m*^*+/−*^: *Cd4*^*+/ΔE4m*^ and *B2m*^*−/−*^: *Cd4*^*ΔE4m/ΔE4m*^ mice are shown. Numbers in left histograms indicate mean fluorescence intensity (MFI)

Of note, a percentage of CD4^+^CD8^−^ cells among the mature thymocyte and lymph node T cell population was decreased in *Cd4*^*ΔE4m/ΔE4m*^ mice (Fig. 2b, f). To examine whether re-directed differentiation of MHC-II selected cells was involved in the decrease of CD4^+^ T cells, as was observed in *Cd4*^*ΔE4p/ΔE4p*^ mice^11^, we analyzed the differentiation of MHC-II selected cells under β2-microglobulin-deficient condition. In CD24^lo^TCRβ^hi^ thymocyte population, while CD4^−^CD8^+^ cells were undetectable in control *B2m*^*−/−*^: *Cd4*^*+/+*^ mice, mature CD4^−^CD8^+^ SP thymocytes emerged in *B2m*^*−/−*^: *Cd4*^*ΔE4m/ΔE4m*^ mice (Fig. 3a, b). In the CD24^hi^TCRb^hi^ population, the percentages of CD8 expressing cells were also higher in *B2m*^*−/−*^: *Cd4*^*ΔE4m/ΔE4m*^ mice (Fig. 3a). As ThPOK is involved in terminating CD8 expression during the maturation of MHC-II selected cells^19,20^, we analyzed *Thpok* expression using the *Thpok*^*gfp*^ reporter allele^16^. Although the majority of MHC-II selected CD24^hi^TCRb^hi^ thymocytes in *B2m*^*−/−*^: *Cd4*^*+/+*^ mice expressed Thpok-gfp at a higher level, biphasic and lower Thpok-gfp expression was observed in those cells in *B2m*^*−/−*^: *Cd4*^*ΔE4m/ΔE4m*^ mice (Fig. 3c), and the CD8 expression level remained higher in Thpok-gfp^lo^ cells than in Thpok-gfp^hi^ cells (Fig. 3c). These observations suggested that a low and delayed CD4 expression after positive selection in the absence of *E4m* resulted in a re-direction of some MHC-II selected thymocytes to the CD8-lineage in part through impaired *Thpok* induction.

However, the percentage of CD4^−^CD8^+^ subset cells declined in the peripheral lymphoid tissues of *B2m*^*−/−*^: *Cd4*^*ΔE4m/ΔE4m*^ mice (Fig. 3b). This might be explained by the differentiation of CD4^−^CD8^+^ thymocytes into CD4^+^CD8^−^ T cells or a defect in the survival of CD4^−^CD8^+^ thymocytes in those mice. To trace CD4 and CD8 expression, we cultured CD4^−^CD8^+^ thymocytes prepared from *B2m*^*−/−*^: *Cd4*^*ΔE4m/ΔE4m*^ mice and found that these cells remained as CD4^−^CD8^+^ cells after five days (Fig. 3d). It has been shown that IL-7 signaling is crucial for the survival and expansion of CD8 SP thymocytes^21,22^. Consistent with a lower IL-7R expression level in the CD4^−^CD8^+^ thymocytes of *B2m*^*−/−*^: *Cd4*^*ΔE4m/ΔE4m*^ mice than in control MHC-I restricted CD4^−^CD8^+^ thymocytes (Fig. 3e), the phosphorylation of Stat5 after in vitro IL-7 stimulation was reduced in *B2m*^*−/−*^: *Cd4*^*ΔE4m/ΔE4m*^ CD4^−^CD8^+^ thymocytes (Fig. 3e). These results suggested that the low level of IL-7R expression in re-directed CD4^−^CD8^+^ thymocytes is involved in the decline in the percentage of CD4^−^CD8^+^ subset in the periphery of *B2m*^*−/−*^: *Cd4*^*ΔE4m/ΔE4m*^ mice.

### Bcl11b is essential for activation of *E4p* and *E4m* enhancers

**Fig. 4:**
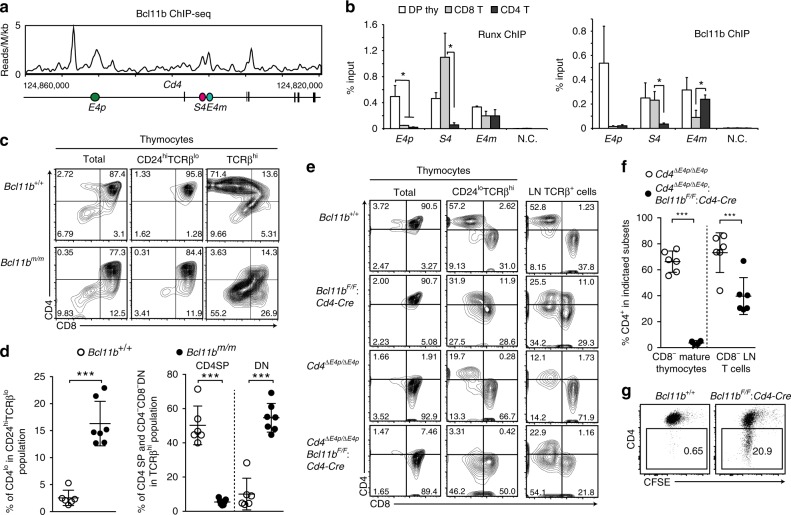
Requirement for Bcl11b in activating *Cd4* at two stages. **a** ChIP-Seq tracks at the *Cd4* gene showing binding of Bcl11b in total thymocytes. **b** ChIP-qPCR analyses for Runx (left) and Bcl11b (right) bindings to the *Cd4* proximal enhancer (*E4p*), *Cd4* silencer (*S4*), and *Cd4* maturation enhancer (*E4m*) in DP thymocytes, peripheral CD4^+^,and CD8^+^ T cells. N.C.: negative control region. Summary of three independent ChIP experiments. Means ± SD. *p < 0.05 (One-way ANOVA and Tukey’s multiple comparison test). **c** Contour plots showing CD4 and CD8 expression in indicated thymocyte subsets of *Bcl11b*^*+/+*^ and *Bcl11b*^*m/m*^ newborn mice. Numbers in quadrants indicate respective cell percentages. Representative results of at least three independent analyses. **d** Summary of percentage of indicated cell types in indicated cell populations of *Bcl11b*^*+/+*^ and *Bcl11b*^*m/m*^ mice. Means ± SD. ***p < 0.001 (unpaired student *t* test, two-sided). **e** Contour plots showing CD4 and CD8 expression in indicated thymocyte subsets and lymph node (LN) T cells of mice with indicated genotypes. Numbers in quadrants indicate respective cell percentages. Representative results of at least three independent analyses. **f** Statistical summary of percentage of CD4^+^ cells in CD8^−^ mature thymocytes and CD8^−^ lymph node (LN) T cells of *Cd4*^*ΔE4p/ΔE4p*^ mice and *Cd4*^*ΔE4p/ΔE4p*^*: Bcl11b*^*F/F*^*: Cd4-Cre* mice mice. Means ± SD. ***p < 0.001 (unpaired student *t* test, two-sided). **g** Dot plots showing CD4 expression and cell divisions five days after in vitro stimulation of sorted CD4^+^ T cells from mice with indicated genotypes. One representative of three experiments

To understand how *E4m* function is regulated, it is necessary to identify molecules that bind and regulate *E4m* activity. During analyses of Bcl11b function^23^, we observed that Bcl11b associates with the *E4p*, *S4*, and *E4m* regions in total thymocytes (Fig. 4a). ChIP-qPCR showed that associations of both Runx and Bcl11b with the *E4p* were dramatically decreased in peripheral T cells (Fig. 4b). Runx binding to *S4* was the highest in CD8^+^ T cells but was almost lost in CD4^+^ T cells, with Bcl11b binding to *S4* also becoming lower in CD4^+^ T cells compared to that in other cell subsets (Fig. 4b). Although the association of Runx to *E4m* was compatible between the three cell subsets, Bcl11b binding to *E4m* was higher in CD4^+^ T cells than in CD8^+^ T cells (Fig. 4b).

Considering such Bcl11b associations with *Cd4* regulatory regions, we examined Bcl11b function in regulating *Cd4* expression by using two *Bcl11b* mutant models. We recently generated a hypomorphic *Bcl11b* allele, referred to as *Bcl11b*^*m*^, that produces truncated Bcl11b protein lacking the last zinc-finger domain^23^. In thymi of newborn *Bcl11b*^*m/m*^ homozygous mice, which die at two days after birth, CD4^lo^CD8^+^ cells were present in the CD24^hi^TCRb^lo^ population, whereas nearly all CD24^hi^TCRb^lo^ thymocytes appeared as CD4^+^CD8^+^ DP cells in control mice (Fig. 4c, d). In addition, CD4 expression in the TCRβ^hi^CD8^−^ mature thymocyte population became lower, resulting in the generation of the CD4^−^CD8^−^ instead of CD4^+^CD8^−^ subset in *Bcl11b*^*m/m*^ thymi (Fig. 4c, d). We next examined the effect of conditional inactivation of *Bcl11b* at DP stage by a *Cd4-Cre* transgene, and observed emergence of the CD4^−^CD8^−^ subset in the CD24^lo^TCRβ^hi^ mature thymocyte population of *Bcl11b*^*F/F*^: *Cd4-Cre* mice (Fig. 4e). In order to further address whether Bcl11b regulates *E4m* activity, we then combined *E4p*-deficiency with *Bcl11b* inactivation. Under an *E4p*-deficient background, the CD4 expression level in the mature CD8-negative thymocyte population severely decreased upon loss of Bcl11b (Fig. 4e, f). In contrast, CD4 expression level was partially restored after egress from the thymus, with CD4^+^ T cells emerging in the peripheral lymphoid tissues of *Cd4*^*ΔE4p/ΔE4p*^: *Bcl11b*^*F/F*^: *Cd4-Cre* mice (Fig. 4e, f). However, the CD4 expression in CD4^+^ T cells that developed in *Bcl11b*^*F/F*^: *Cd4-Cre* mice were unstable after activation-induced cell divisions (Fig. 4g). Thus, dysfunction of Bcl11b resulted in a delayed CD4 induction at two transitional stages, one for becoming DP thymocytes and the other for becoming mature CD4 SP thymocytes, which require activation of *E4p* and *E4m*, respectively.

### Characterizing specific activity of *E4m*

**Fig. 5:**
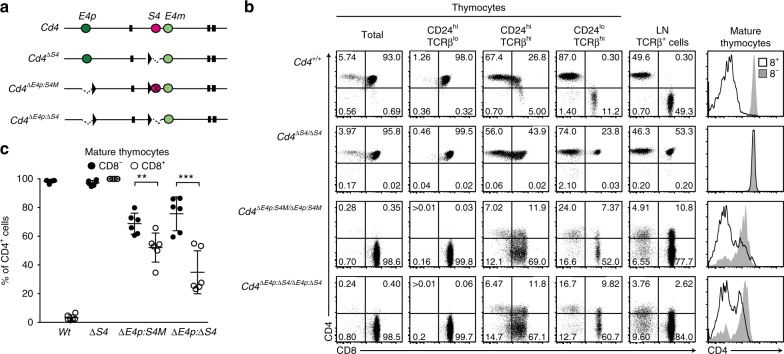
Helper-lineage dominant activity of *E4m*. **a** Schematic structures of *Cd4*, *Cd4*^*ΔS4*^, *Cd4*^*ΔE4p:S4M*^, and *Cd4*^*ΔE4p:ΔS4*^ alleles are shown as in Fig. 2a. Mutations at two Runx sites within *S4* are marked as X in the *Cd4*^*ΔE4p:S4M*^ allele. **b** Dot plots showing CD4 and CD8 expression in indicated thymocyte subsets of mice with indicated genotypes. Numbers in quadrants indicate respective cell percentages. Right histograms showing CD4 expression in CD8^+^ and CD8^−^ mature (CD24^lo^TCRβ^hi^) thymocyte populations. Representative results of at least five independent analyses. **c** Summary of percentage of CD4^+^ cells in CD8^−^ and CD8^+^ mature (CD24^lo^TCRβ^hi^) thymocyte populations of *Cd4*^*+/+*^ (*Wt*), *Cd4*^*ΔS4/ΔS4*^ (^*Δ*^*S4*), *Cd4*^*ΔE4p:S4M/ΔE4p:S4M*^ (*ΔE4p:S4M*), and *Cd4*^*ΔE4p:ΔS4/ΔE4p:ΔS4*^ (Δ*E4p:ΔS4*) mice. Means ± SD. **p < 0.01, ***p < 0.001 (unpaired student *t* test, two-sided)

In order to further characterize *E4m* activity, we addressed how *E4m* alone behaves by generating a *Cd4*^*ΔE4p:ΔS4*^ allele, in which only the *E4m* among three regulatory regions remains in the *Cd4* gene (Fig. 5a). Notably, the percentage of cells expressing CD4 was higher in CD8-negative cells than in CD8-positive cells in the mature thymocyte population of *Cd4*^*ΔE4p:ΔS4/ΔE4p:ΔS4*^ mice (Fig. 5b, c), although it was equally high in both types of cells of *Cd4*^*ΔS4/ΔS4*^ mice. To exclude a possibility that deleted sequences around *S4* in the *Cd4*^*ΔE4p:ΔS4*^ allele include a functional element for *E4m* activity, we targeted specific mutations onto two Runx-motifs within *S4* to abrogate *S4* function by a more specific manner^9^, generating a *Cd4*^*ΔE4p:S4M*^ allele (Fig. 5a). In *Cd4*^*ΔE4p:S4M/ΔE4p:S4M*^ mice, the percentage of cells expressing CD4 remained higher in CD8^−^ cells compared to CD8^+^ T cells (Fig. 5b, c). Such CD8^+^ cells were almost undetected in *B2m*^*−/−*^: *Cd4*^*ΔE4p:S4M/ΔE4p:S4M*^ mice (Supplementary Fig. 4), indicating that the majority of CD8^+^ cells in *Cd4*^*ΔE4p:S4M/ΔE4p:S4M*^ mice constituted MHC-I restricted cells. Given that CD4 expression from the *Cd4*^*ΔE4p:ΔS4*^ allele is likely to depend on *E4m*, these observations indicate that the *E4m* activity is more predominant in the helper-lineage cells than in cytotoxic-lineage cells.

### Regulation of *E4m* activity by Runx complexes

**Fig. 6:**
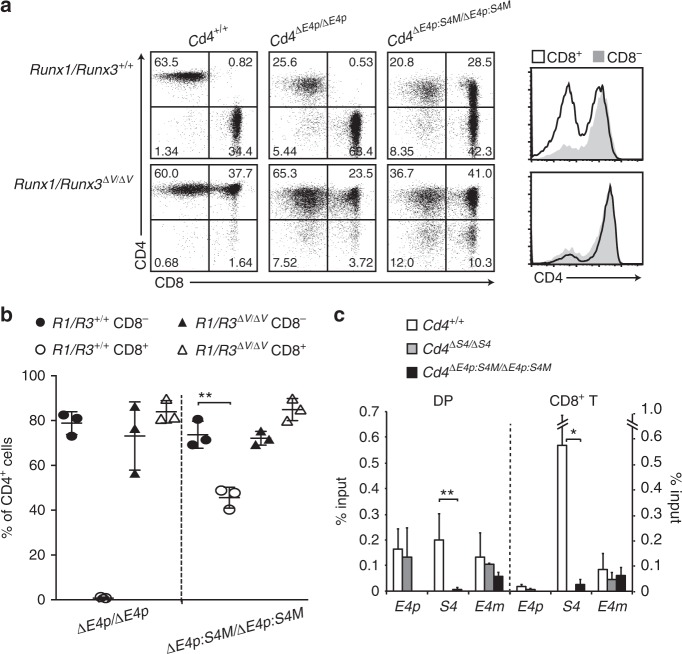
Involvement of Runx in regulating the helper-lineage dominant activity of *E4m*. **a** Dot plots showing CD4 and CD8 expression in lymph node T cells of mice with indicated genotypes. In *Runx1/Runx3*^*ΔV/ΔV*^ mice, both Runx1 and Runx3 proteins lack the VWRPY motif, which is essential for interaction with TLE/Groucho co-repressors. Right histograms showing CD4 expression level in CD8^−^ and CD8^+^ lymph node T cells of *Cd4*^*ΔE4p:S4M/ΔE4p:S4M*^ mice with (lower) or without (upper) *Runx1/Runx3*^*ΔV/ΔV*^ mutation. Numbers in quadrants indicate respective cell percentages. One representative result of three independent analyses. **b** Summary of percentage of CD4^+^ cells in CD8^−^ and CD8^+^ lymph node T cells of *Cd4*^*ΔE4p/ΔE4p*^, *Cd4*^*ΔE4p:S4M/ΔE4p:S4M*^ mice with or without *Runx1/Runx3*^*ΔV/ΔV*^ mutation. Means ± SD. **p < 0.01 (unpaired student *t* test, two-sided). **c** Graph showing summary of three independent ChIP-qPCR for Runx bindings to *E4p*, *S4*, and *E4m* regions in DP thymocytes (DP) and CD8^+^ T cells from *Cd4*^*+/+*^, *Cd4*^*ΔS4/ΔS4*^ and *Cd4*^*ΔE4p:S4M/ΔE4p:S4M*^ mice. Means ± SD. *p < 0.05, **p < 0.01 (One-way ANOVA and Tukey’s multiple comparison test)

To examine how such helper-lineage dominant activity of *E4m* is regulated, we analyzed the function of transcription factors that are known to be involved in lineage-specific expression of the *Cd4* gene. Two Runx proteins, Runx1 and Runx3, are essential to control lineage specific *Cd4* expression through activating the *S4* silencer^9^. The penta-peptide sequences, VWRPY, at the C-terminal end of Runx proteins serve as a platform to recruit the TLE/Groucho co-repressor family^24,25^, and are essential for Runx-mediated *Cd4* repression^10,26^. Accordingly, mice lacking the VWRPY motif from both Runx1 and Runx3 proteins (*Runx1/Runx3*^*ΔV/ΔV*^ mice), exhibited full CD4 de-repression in CD8^+^ T cells, phenocopying the *Cd4*^*ΔS4/ΔS4*^ mice (Figs 5b and 6a). Given a loss of *S4* activity by *Runx1/Runx3*^*ΔV/ΔV*^ mutation, we assumed that CD4 expression in *Runx1/Runx3*^*ΔV/ΔV*^*: Cd4*^*ΔE4p/ΔE4p*^ mice was similar to that in *Cd4*^*ΔE4p:ΔS4/ΔE4p:ΔS4*^ mice. However, the percentage of CD4-expressing cells was comparable between CD8^+^ and CD8^−^ cells in *Runx1/Runx3*^*ΔV/ΔV*^*: Cd4*^*ΔE4p/ΔE4p*^ mice (Fig. 6a, b). Thus, a discrepancy existed between the effects from loss of *cis*-acting *S4* and loss of *trans-*acting Runx proteins on the *Cd4*^*ΔE4p*^ allele, suggesting a possible involvement of *S4*-indpendent mechanisms through which Runx proteins suppress *E4m* activity. We then generated *Runx1/Runx3*^*ΔV/ΔV*^*: Cd4*^*ΔE4p:S4M/ΔE4p:S4M*^ mice and observed an increase of the CD4 expressing population in CD8^+^ T cells (Fig. 6a, b). Our ChIP-qPCR analyses showed that Runx association with the *E4m* was unaffected by eliminating Runx-motifs in *S4*, whereas Runx binding to *S4* was abrogated (Fig. 6c). Together, these observations indicated that Runx proteins are likely to repress *E4m* activity in a VWRPY-dependent manner through their *S4*-independent association with *E4m*.

Lack of canonical Runx-motifs within *E4m* suggests that recruitment of Runx complexes to *E4m* might be mediated via protein-protein interaction. As both Bcl11b and Satb1 are functional activators for the *E4m* enhancer and interact with Runx proteins^23,27^, we wished to test whether Runx association with *E4m* is affected by lack of Bcl11b and Satb1. However, the severe reduction of mature thymocytes in *Bcl11b*^*F/F*^*: Satb1*^*F/F*^*: Cd4-Cre* mice (Supplementary Fig. 5a) made it possible to perform ChIP-qPCR analyses only in DP thymocytes. In this setting, Runx binding to *S4*, *E4p*, and *E4m* was not significantly changed by the loss of Bcl11b and Satb1proteins (Supplementary Fig. 5b), suggesting the presence of another factor(s) that recruits Runx complexes to *E4m*.

### Repressive regulation of *E4m* by ThPOK

**Fig. 7:**
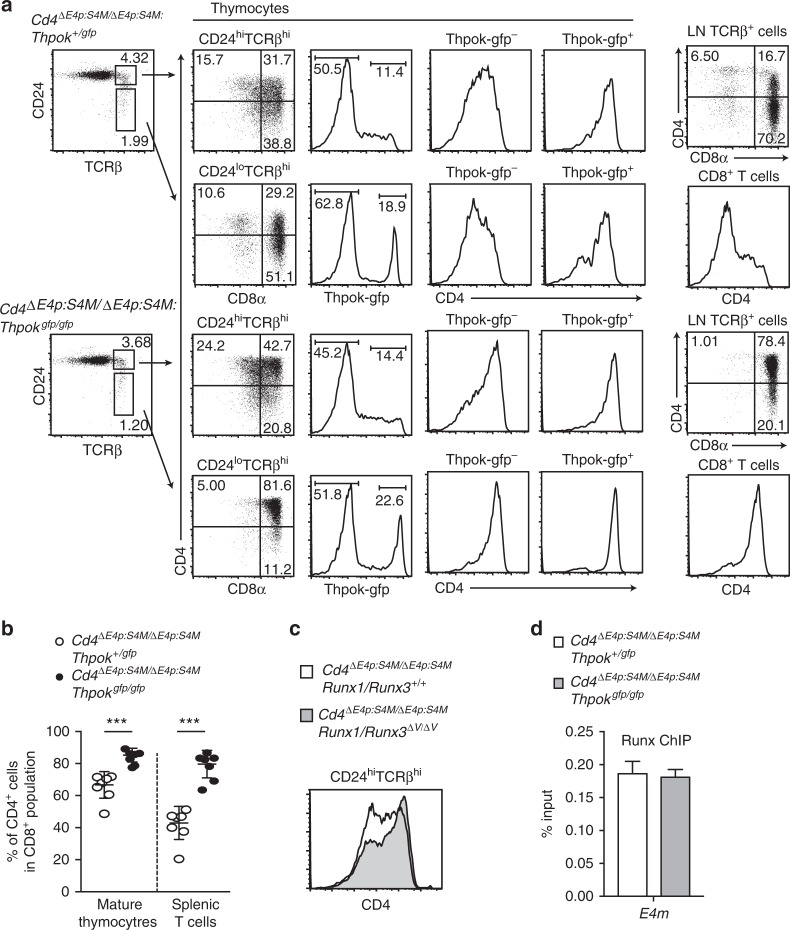
ThPOK is necessary for Runx-mediated repression of *E4m* activity. **a** Flow cytometry analyses for CD4, CD8 and Thpok-gfp expression during T cell development in *Cd4*^*ΔE4p:S4M/ΔE4p:S4M*^ mice in the presence or absence of ThPOK. Numbers in quadrants, indicated gate, and the region in histograms indicate respective cell percentages. **b** Summary of percentage of CD4^+^ cells in CD8^+^ mature thymocytes and CD8^+^ splenic T cells of *Cd4*^*ΔE4p:S4M/ΔE4p:S4M*^*: Thpok*^*+/gfp*^ and *Cd4*^*ΔE4p:S4M/ΔE4p:S4M*^*: Thpok*^*gfp/gfp*^ mice. Means ± SD. *** *p* < 0.001 (unpaired student *t* test, two-sided). **c** Histogram showing CD4 expression in freshly selected (CD24^hi^TCRβ^hi^) thymocytes of *Cd4*^*ΔE4p:S4M/ΔE4p:S4M*^*: Runx1/Runx3*^*+/+*^ and *Cd4*^*ΔE4p:S4M/ΔE4p:S4M*^*: Runx1/Runx3*^*ΔV/ΔV*^ mice. One representative of two independent analyses. **d** Graph showing summary of three independent ChIP-qPCR experiments for Runx bindings to *E4m* in pre-selection DP thymocytes from *Cd4*^*ΔE4p:S4M/ΔE4p:S4M*^*: Thpok*^*+/gfp*^ and *Cd4*^*ΔE4p:S4M/ΔE4p:S4M*^*: Thpok*^*gfp/gfp*^ mice. Means ± SD

As ThPOK was shown to be essential for the regulation of lineage-specific CD4 expression by counteracting against *S4*-mediated *Cd4* repression^16,28^, we next examined whether ThPOK is involved in the regulation of *E4m* activity by generating *Cd4*^*ΔE4p:S4M/ΔE4p:S4M*^*:Thpok*^*gfp/gfp*^ mice that lack ThPOK expression. Notably, loss of ThPOK resulted in an increase of CD4 expressing cells in mature CD8^+^ thymocyte and peripheral CD8^+^ T cell populations (Fig. 7a, b). Increase of the CD4^+^ subset by lack of ThPOK had already been observed at the CD24^hi^TCRβ^hi^ stage composed of freshly selected thymocytes and in Thpok-gfp^−^ cells that represent MHC-I-signaled ThPOK non-expressing cells. These genetic results suggested that ThPOK restrains *E4m* activity from the early initiation phase. Similar early induction of CD4 in CD24^hi^TCRβ^hi^ thymocytes from the *Cd4*^*ΔE4p:S4M*^ allele was also observed by *Runx1/Runx3*^*ΔV/ΔV*^ mutation (Fig. 7c), suggesting that the mechanism by which Runx represses *E4m* activity became non-functional through the lack of ThPOK expression. Our ChIP-qPCR using peripheral CD4^+^ T cells showed that ThPOK associated with *E4m* in the absence of the *S4* (Supplementary Fig. 5c), and Runx association with *E4m* in pre-selection DP thymocytes was not affected by loss of ThPOK (Fig. 7d). Interestingly, level of H3K27ac at *E4m* region tended to be higher in CD8^±^ mature thymocytes in the absence of ThPOK, although it was lower than that in control CD4 SP thymocytes (Supplementary Fig. 5d). These observations unraveled an unexpected ThPOK function that assists Runx-mediated repression of *E4m* activity after recruitment of Runx to the *E4m* enhancer.

## Discussion

By generating several mutant *Cd4* alleles, our study provides novel insights into how two enhancers, *E4p* and *E4m*, regulate *Cd4* expression (Supplementary Fig. 6). *E4p* and *E4m* cooperate to establish a stably heritable active state in the helper-lineage T cells by activating the DNA de-methylation process. It is unclear whether such property is endowed to specific enhancers or is a general feature of enhancers. We showed that a synthetic *Eth* enhancer derived from the *Thpok* gene could compensate the *E4p* activity that induces DNA de-methylation. This suggests that specific sequences within the *E4p* are unlikely to be absolutely essential to control DNA de-methylation at the *Cd4* locus. Despite their differences in sequences, it is possible that *Eth* and *E4p* poise similar epigenetic codes that can be recognized by common machinery that induces DNA de-methylation. Recent work reported accumulation of 5hmC, which is generated by TET family proteins through DNA de-methylation processs^29^, near the *Thpok* proximal enhancer core region in the CD4 SP thymocytes^30^, suggesting that TET proteins are involved in DNA de-methylation for activation of the *Thpok* gene. Thus, once distinct protein complexes bound on a distinct enhancer marks the target locus with such epigenetic codes, a sequential reaction that induces DNA de-methylation might be carried out. Alternatively, *E4p/E4m* enhancers and *Eth* were bound by same nuclear protein(s) that recruit machinery to induce DNA de-methylation. Bcl11b and Satb1 may represent candidates for such shared factors, as both proteins are involved in the activation of *E4m* in the *Cd4* gene and the *PE* enhancer in the *Thpok* gene^23,27^. This also suggests that activation of the *Thpok* gene might involve DNA de-methylation. Release of *Thpok* silencing from CD8^+^ T cells by an inhibitor for maintenance methyltransferase^31^ and the low *Thpok* expression level effected by a lack of Tet2/3 proteins^15^ support the involvement of DNA de-methylation in *Thpok* activation.

In *B2m*^*−/−*^: *Cd4*^*ΔE4m/ΔE4m*^ mice, a re-directed CD4^−^CD8^+^ cell subset is generated in the thymus in part due to impaired ThPOK induction; however, these CD4^−^CD8^+^ thymocytes failed to expand sufficiently to generate a visible population in the periphery. As these cells are MHC-II restricted, the lack of TCR signaling by loss of CD4 expression could impair their survival capacity^32^. However, previous reports have shown that mice expressing a low level of ThPOK retain re-directed CD4^−^CD8^+^ MHC-II restricted cells in the periphery^16,33^. It is possible that aberrant CD4 expression kinetics unique to *Cd4*^*ΔE4m/ΔE4m*^ mice may generate CD4^−^CD8^+^ MHC-II restricted cells that acquire IL-7-dependent survival capacity similar to normal MHC-I restricted CD8 SP thymocytes^21,22^ with a low level of IL-7Ra expression. An imbalance between attaining an IL-7-responsive property and inefficient receptor expression might thus inhibit the expansion of CD4^−^CD8^+^ MHC-II restricted cells in *Cd4*^*ΔE4m/ΔE4m*^ mice.

Our genetic results clearly showed that Runx proteins are necessary for *S4*-independent repression of *E4m* activity in CD8^+^ T cells. Previous research showed that mutant Runt protein, a counterpart of Runx protein in *Drosophila*, lacking DNA binding activity by point mutations in the Runt-domain, retains the activity to repress the segment polarity gene *engrailed* (*en*)^34^, indicating that recognition of the Runx-motif is not absolutely necessary for Runx-mediated repression. Once Runx proteins are included into nuclear protein complexes on *E4m*, TLE/Groucho co-repressor family proteins are likely to be recruited and suppress *E4m* activity in a CD8-lineage-predominant manner. In contrast, although Runx complexes also associate with the *E4p* enhancer even in the absence of *S4*, inhibition of *E4p* activity requires *S4* sequences. Thus, the modes of Runx action to repress the two enhancers in the *Cd4* locus are different, although the VWRPY motif in Runx proteins are shared by two modes. It is possible that protein complexes formed on the *S4* region are required specifically to repress transcriptional activators on the *E4p* enhancer. Alternatively, based on the different position of the two enhancers with respect to *S4*, topological regulation such as prevention of chromatin looping between *E4p* and the *P4* promoter may require *S4*-dependent regulation.

An *S4*-independent regulation of lineage specificity of *E4m* activity challenges the previous silencer-based model that has been proposed to explain how lineage-specific *Cd4* expression is regulated. Association of Runx with *E4m* in both cytotoxic-lineage and helper-lineage cells suggests that Runx-mediated *E4m* repression is likely to be canceled in a helper-lineage-specific manner. ThPOK was shown to antagonize *S4* activity to maintain CD4 expression in helper-lineage cells^16,28^. However, our genetic results indicate that ThPOK instead assists Runx-mediated *E4m* repression. It remains elusive how ThPOK exerts such opposite regulation against *S4*-dependent and -independent Runx function. Considering that loss of ThPOK expression affects CD4 expression from the *Cd4*^*ΔE4p:S4M*^ allele in freshly selected thymocytes through MHC-I, it is worth taking into account the possibility that ThPOK expression in early hematopoiesis is involved in later regulation of *E4m* activity. Recruitment of HDACs was proposed to be a possible mechanism by which ThPOK repress *Cd8* gene^19^. Along with a higher H3K27ac status at *E4m* region in ThPOK-deficient cells, ThPOK might regulate *E4m* activity via histone acetylation. Alternatively, considering that ThPOK has been shown to be involved in tethering genomic regions to the nuclear lamina to effect gene repression^35^, it is also possible to ThPOK regulate *Cd4* expression by altering nuclear positioning of the *Cd4* locus during T cell development. Regardless, our genetic results shed new light on the roles of Runx and ThPOK in *Cd4* gene regulation by regulating a novel *E4m* enhancer.

## Methods

### Generation of mutant *Cd4* alleles

In order to generate the target vector for the *Cd4*^*Eth*^ allele, we first generated an *Eth* DNA fragment by overlap PCR. In the *Eth* sequences, core enhancer sequences from the thymic enhancer (*TE*) and the proximal enhancer (*PE*) from the *Thpok* locus were conjugated (Supplementary Fig. 1a). In order to replace *E4p* with *Eth*, the *Eth* DNA fragment was ligated into the *Sal*I site of the target vector (a gift from Mark Chong) that was used to generate the *E4p*^*Δ/Δ*^ mice^11^. Transfection of target vectors into M1 ES cells and identification of ES clone underwent homologous recombination by PCR were performed. During a process of removal of the neomycin resistant gene *(neo*^*r*^*)* by transient transfection of a Cre expression vector, pMC-Cre, ES clones harboring *Cd4*^*+/Eth*^ or *Cd4*^*+/ΔE4p*^ genotype were generated from the into ES clone harboring the *Cd4*^*+/EthN*^ genotype.

In order to make the target vector for the *Cd4*^*ΔE4m*^ allele in which mouse genomic region corresponding to 124835251-124835615 in NCBI37/mm9 were deleted (Supplementary Fig. 2b), we first amplified the 124834833-1283251 region by PCR to add BglII site at 5′ end and SpeI site at 3′ end. In order to make a longer 3′ homology region, a DNA fragment prepared from the pCassette vector^36^ by SpeI/KpnI digestion was ligated to the 3′ end. A DNA fragment that harbors 5′ homology region, the *neo*^*r*^ gene and the *Cd4* silencer region was prepared from the pCassette vector by HindII/BglII digestion, and were ligated together with a BglII/KpnI 3′ homology region into the HindIII/KpnI cleaved pUC18 vector. In order to generate ES clones harboring the *Cd4*^*+/ΔE4m*^ and *Cd4*^*+/ΔE4p:ΔE4m*^ genotype, the target vector for the *Cd4*^*ΔE4m*^ allele was transfected into ES clone harboring *Cd4*^*ΔS4/ΔE4p*^ genotype. After isolating ES clones that underwent homologous recombination, we examined which allele, *Cd4*^*+*^ or *Cd4*^*ΔE4p*^, was targeted by retroviral Cre transduction, followed by PCR that distinguished pattern of recombination between three loxP sites (Supplementary Fig. 2c). ES clones harboring the *Cd4*^*+/ΔS4*^ or *Cd4*^*+/ΔE4p:ΔS4*^ were similarly generated by transfection of the target vector that remove *S4* core region (124835896–124836384 in NCBI37/mm9) into *Cd4*^*+/+*^ or *Cd4*^*+/Eth*^ ES clone, respectively. ES clones harboring the *Cd4*^*+/ΔE4p:S4M*^ were generated by transfection of the target vector, which was used to target mutation into the Runx sites within the *S4*^9^, into the *Cd4*^*+/ΔE4p*^ ES clone.

### Mice

*Runx1*^*ΔV*^ mice^37^, *Runx3*^*ΔV*^ mice^38^, *Thpok*^*gfp*^ mice^16^ and *Bcl11b*^*F*^ and hypomorphic *Bcl11b*^*m*^ mice^23^ have been described. β2m-deficient mice (002087) were purchased from the Jackson laboratory. *Cd4*^*+/ΔLS*^ mice were generated by crossing *Cd4*^*+/Sflox*^ mice^6^ (a gift from Dr. D. R. Littman) to the *EIIa-Cre* mouse (003724) from the Jackson Laboratory. Generation of chimera mice from ES cells by aggregation was performed by the animal facility at RIKEN, IMS. All mice were maintained in the animal facility at the RIKEN IMS, and all animal procedures were in accordance with protocol approved by the institutional Animal Care and Use Committee (IACUC) of RIKEN Yokohama Branch.

### Flow cytometry analyses and cell sorting

Single cell suspensions from thymus, spleen and lymph nodes were prepared by mashing tissues through a 70 μm cell strainer (BD Bioscience). Single cell suspensions were stained with following antibodies purchased from BD Bioscience or eBiosciences: CD4 (25-0042-81: eBiosciences, RM4-5), CD8α (553035, 563068: BD Biosciences, 53-6.7), CD24 (562563: BD Biosciences, M1/69), CD69 (561931: BD Biosciences, H1.2F3), IL7Rα (17-1271-82: eBiosciences, A7R34) and TCRβ (47-5961-82: eBiosciences, H57-597). For intracellular staining of phosphorylated Stat5 (612599: BD Biosciences, 47), cells were stained with cell surface molecules and were stimulated recombinant mouse IL-7 (R&D) at the indicated time and concentration. After stimulation, cells were fixed and permeabilized with BD Phosflow™ Lyse/Fix buffer (558049: BD Biosciences) and Perm Buffer III (558050: BD Biosciences). Antibodies were used at a concentration of 2.5 μg ml^−1^. Multi-color flow cytometry analysis was performed using a BD FACSCanto II (BD-Bioscience) and data were analyzed using FlowJo (Tree Star) software. Cell subsets were sorted using a BD FACSAria II (BD Biosciences).

### DNA methylation analyses

DNA purified from FACS-sorted cells was subjected to bisulfite reactions with MethylEasy™ Xceed (Genetic Signatures) according to the manufacturer’s protocol. PCR product amplified from this DNA was cloned into the pCR^TM^II Vector using a TA Cloning® Kit (ThermoFisher scientific) and was sequenced. The following primer set was used for PCR amplification: Cd4R1F, 5′-TTGATTTTTTAAAATAGAAAGGTTT-3′ and Cd4R1R, 5′-AAATATCTAAAATATACCAACCACTAC-3′.

### Quantitative RT-PCR

DNase I-treated total RNA was prepared from sorted cells using RNeasy mini kit (QIAGEN) and cDNA was synthesized by SuperScript™ IV reverse transcriptase (ThermoFisher SCIENTIFIC). Quantitative RT-PCR was performed using QuantStudio™ 3 Real-Time PCR system (Applied Biosystems) with Universal ProbeLibrary (Roche). The following primer sets and probes were used for *Cd4* and *Hprt* mRNA quantification: Cd4-F, 5′-TCTGGAACTGCACCGTGAC-3′, Cd4-R, 5′-CCGTGATAGCTGTGCTCTGA-3′ and UPL #93. Hprt-F, 5′-TCCTCCTCAGACCGCTTTT-3′, Hprt-R, 5′-CCTGGTTCATCATCGCTAATC-3′ and UPL #95.

### Chromatin immune-precipitation (ChIP)

ChIP-Seq data for Runx/Cbfβ complexes and Bcl11b binding in total thymocytes were taken from existing deposited data (GEO accession number: GSE90949 and GSE90794, respectively). For analytical ChIP-qPCR, FACS-sorted or MACS (Mitenyi Biotec)-separated 0.5 to 3 × 10^7^ cells were used to prepare chromatin DNA. Cells were cross-linked by incubation in a 1% of formaldehyde solution for 10 min with gentle rotation at room temperature. Nuclei were separated and were sonicated using a model XL2000 ultrasonic cell disruptor (MICROSON). Sonicated chromatin was incubated overnight at 4 °C with anti-Histone H3K27ac (clone D5E4, Cell Signaling Technology), anti-Histone H3K27me3 (clone C36B11), 5 ug of anti-Cbfβ rabbit polyclonal antibody^39^, anti-Bcl11b antibody (A300-385A, Bethyl Laboratories) or anti-ThPOK polyclonal antibody^23^ that were pre-conjugated with Dynabeads M-280 Sheep anti-Rabbit IgG (Thermo Fisher Scientific). After washing beads, immunoprecipitates were eluted from beads into elution buffer. Eluted immunoprecipitates were then incubated at 65 °C overnight for reverse-crosslinking. Input DNA and ChIP DNA were treated with RNaseA (Thermo Fisher Scientific) and Proteinase K (Thermo Fisher Scientific), and then were purified by ChIP DNA Clean and Concentrator^TM^ kit (ZYMO RESEARCH). Quantitative PCR was performed using the QuantStudio™ 3 Real-Time PCR system (Applied Biosystems) with SYBR Green detection system. Primers sequences for quantitative PCR are listed in the Supplementary Table 1.

### In vitro T-cell culture

Sorted CD4^+^ T cells were labeled with CFSE (ThermoFisher Scientific) and were cultured in custom ordered Dulbecco’s Modified Eagle Medium (D-MEM, KOHJIN BIO) supplemented with 10% heat inactivated FBS (Hyclone). 2.0 × 10^5^ cells were stimulated in 96-well flat-bottomed plate, which was pre-coated with 2 μg ml^−1^ anti-CD3e antibody (553068, BD Bioscience) and 2 μg ml^-1^ soluble anti-CD28 antibody (553295, BD Bioscience), for two days, and were maintained in D-MEM supplemented with 10 ng ml^-1^ recombinant mouse IL-2 (402-ML, R&D system) for 2 to 4 days.

### Data availability

ChIP-seq data that support the findings of this study have been deposited with accession GSE90949 and GSE90794, respectively. All other relevant data are available from the authors.

## Electronic supplementary material

Supplementary Information
