# Supplementary material for: Runx-dependent and silencer-independent repression of a maturation enhancer in the Cd4 gene
Source: Nat Commun. 2018 Sep 5;9:3593. doi: 10.1038/s41467-018-05803-3 (PMC6125603; doi:10.1038/s41467-018-05803-3)
Supplement: Supplementary file 1 — Supplementary Information [file 41467_2018_5803_MOESM1_ESM.pdf]

Runx-dependent and silencer-independent repression of a maturation enhancer in the *Cd4* gene

Kojo S, et al.

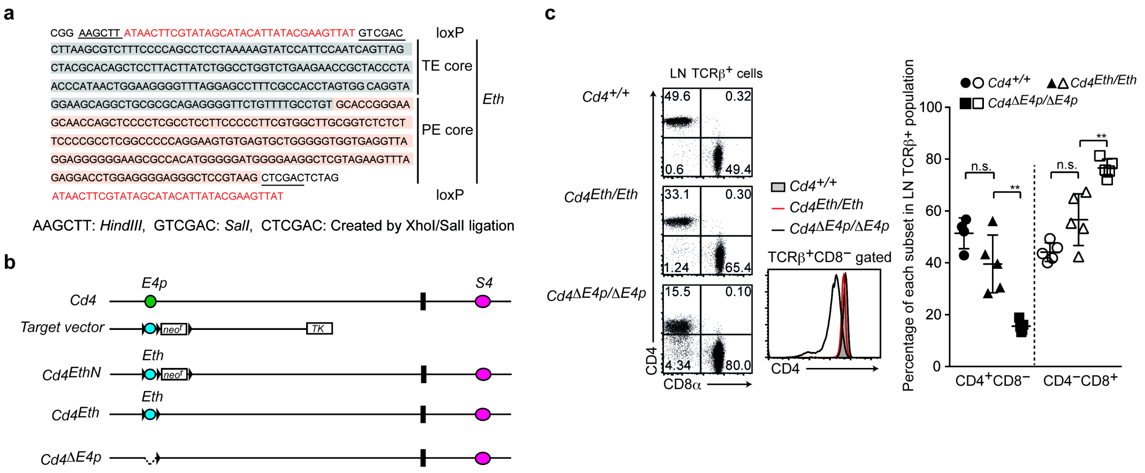

Supplementary Figure 1. Generation of a *Cd4*^{Eth} allele. (**a**) Sequence information of an *Eth* fragment in the *Cd4*^{Eth} allele. Sequences derived from *Thpok* thymic enhancer (*TE*) and *Thpok* proximal enhancer (*PE*) is shaded with gray and orange color. The loxP sequences are shown in red font. (**b**) Schematic structures of *Cd4* gene, target vector and targeted mutant *Cd4* alleles. Ovals marked with different colors represent *cis*-regulatory regions, *Cd4* proximal enhancer (*E4p*), synthetic *Thpok* enhancer (*Eth*) and *Cd4* silencer (*S4*). Black box and triangle are exons and loxP sequences, respectively. *neo*^{r}: neomycin resistant gene, *TK*: HSV thymidine kinase gene. (**c**) Dot plots showing CD4 and CD8 expression in lymph node (LN) TCRβ^{+} cells and histograms at the middle showing CD4 expression in TCRβ^{+}CD8^{-} LN cells from *Cd4*$^{+/+}$, *Cd4*$^{Eth/Eth}$ and *Cd4*$^{\Delta E4p/\Delta E4p}$ mice. Numbers in quadrants indicate respective cell percentages. Graph at right showing summary of percentage of CD4^{+}CD8^{-} and CD4^{-}CD8^{+} subsets in *Cd4*$^{+/+}$, *Cd4*$^{Eth/Eth}$ and *Cd4*$^{\Delta E4p/\Delta E4p}$ mice. Means ± SD. ** $P < 0.01$ (unpaired student *t* test).

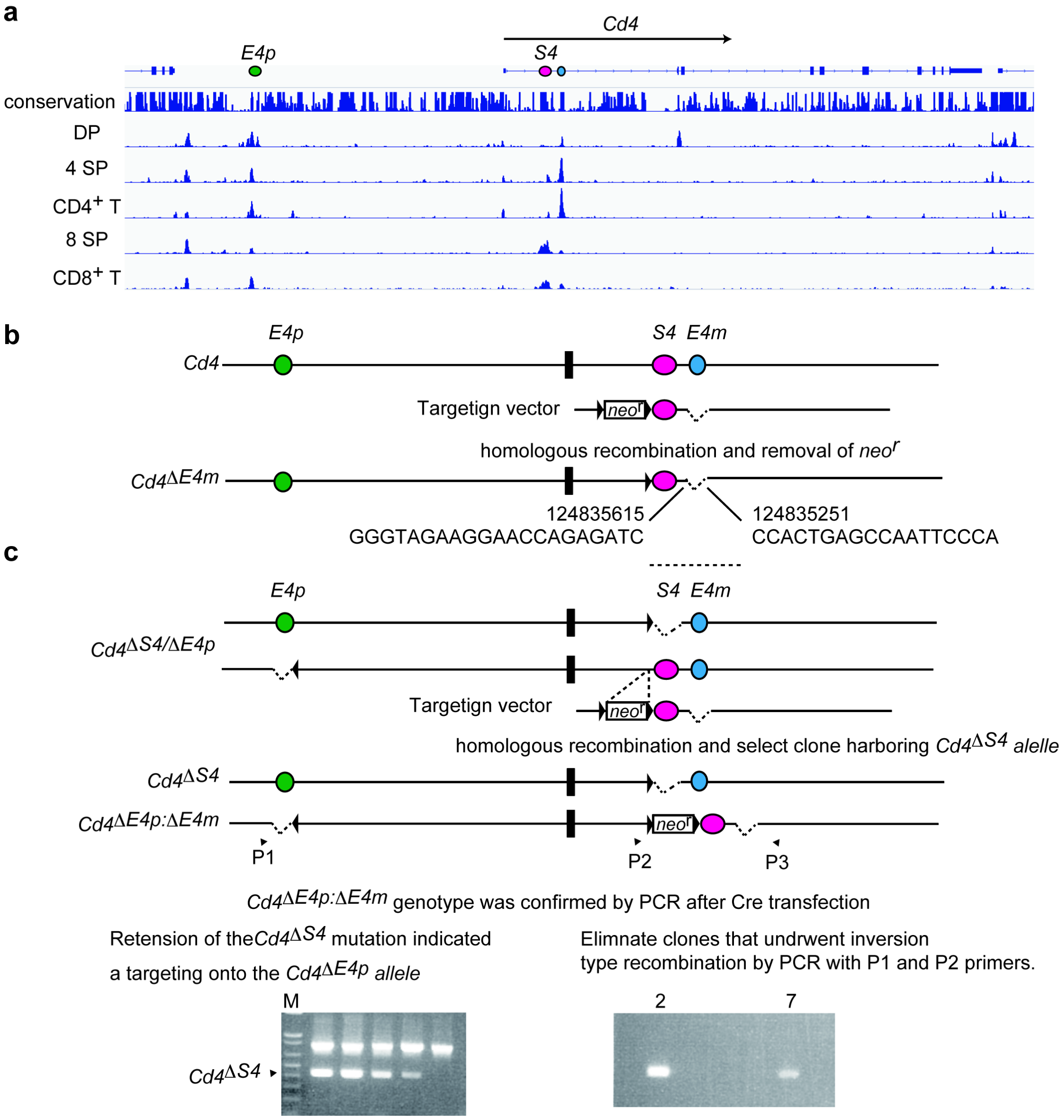

Supplementary Figure 2. Generation of *Cd4*$^{\Delta E4m}$ and *Cd4*$^{\Delta E4p:\Delta E4m}$ alleles. (**a**) ATAC-seq track around the murine *Cd4* gene is shown with conservation. ATAC-seq results in CD4^{+}CD8^{+} DP thymocytes (DP), CD4 single positive thymocytes (4SP), spleen CD4^{+} naive T cells (CD4^{+} T), CD8 single positive thymocytes (8SP) and spleen CD8^{+} naive T cells (CD8^{+} T) are from ImmGen database. Positions of *Cd4* proximal enhancer (*E4p*) and *Cd4* silencer (*S4*) are marked with green and red ovals, respectively. (**b**) Schematic structures of *Cd4* gene, target vector and mutant *Cd4*$^{\Delta E4m}$ allele. Ovals marked with different colors represent *Cd4* proximal enhancer (*E4p*), *Cd4* silencer (*S4*),

and *Cd4* maturation enhancer (*E4m*). Black box and triangle are exons and loxP sequences, respectively. *neo*^{r}: neomycin resistant gene. (**c**) Schematic structures of $Cd4^{\Delta S4}$ and $Cd4^{\Delta E4p}$ alleles. Ovals marked with different colors represent *Cd4* proximal enhancer (*E4p*), Cd4 silencer (*S4*) and *Cd4* maturation enhancer (*E4m*). The target vector was transfected into ES clone harboring $Cd4^{\Delta S4/\Delta E4p}$ genotype. After identification of ES clones that underwent homologous recombination with the target vector, presence or absence of the $Cd4^{\Delta S4}$ allele was examined by PCR with P2 and P3 primers to examine which allele, $Cd4^{\Delta S4}$ or $Cd4^{\Delta E4p}$, underwent homologous recombination. After transient transfection of Cre expression vector, G418-sensitive clones were analyzed by PCR with appropriate primer sets to conform removal of the *neo*^{r} gene and to eliminate clones that underwent inversion type recombination.

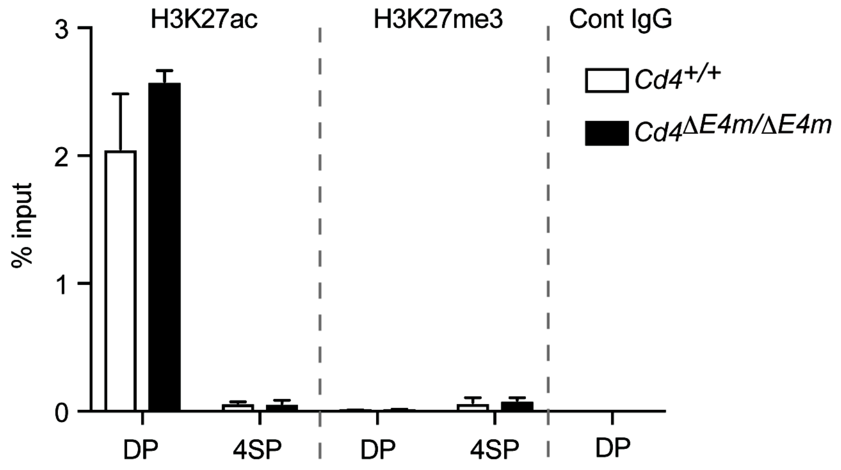

Supplementary Figure 3. Histone modifications at the *E4p* enhancer in the absence of *E4m* enhancer. Levels of histone modifications (H3K27ac and H3K27me3) at the *Cd4* proximal enhancer (*E4p*) in $CD4^{+}CD8^{+}$ double positive (DP) and mature CD4 single positive (4SP) thymocytes from $Cd4^{+/+}$ and $Cd4^{\Delta E4m/\Delta E4m}$ mice are shown. Means ± SD. (*n*=3 per group).

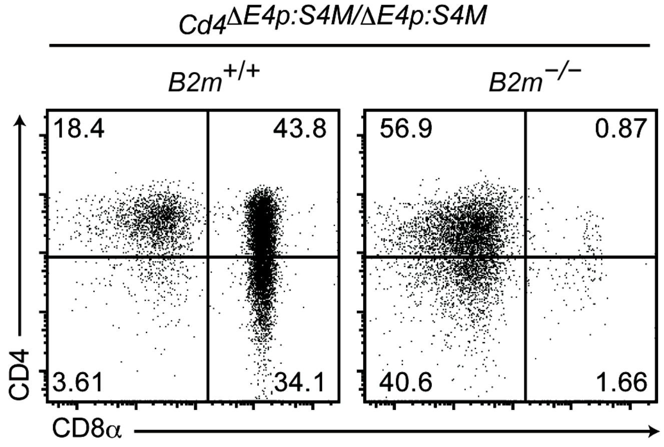

Supplementary Figure 4: MHC-I restriction of CD8^{+} T cells emerged in *Cd4*$^{\Delta E4p:S4M/\Delta E4p:S4M}$ mice. Dot plots showing CD4 and CD8 expression in lymph node T cells of *B2m*$^{+/+}$: *Cd4*$^{\Delta E4p:S4M/\Delta E4p:S4M}$ and *B2m*$^{-/-}$: *Cd4*$^{\Delta E4p:S4M/\Delta E4p:S4M}$ mice. One representative of two independent analyses is shown.

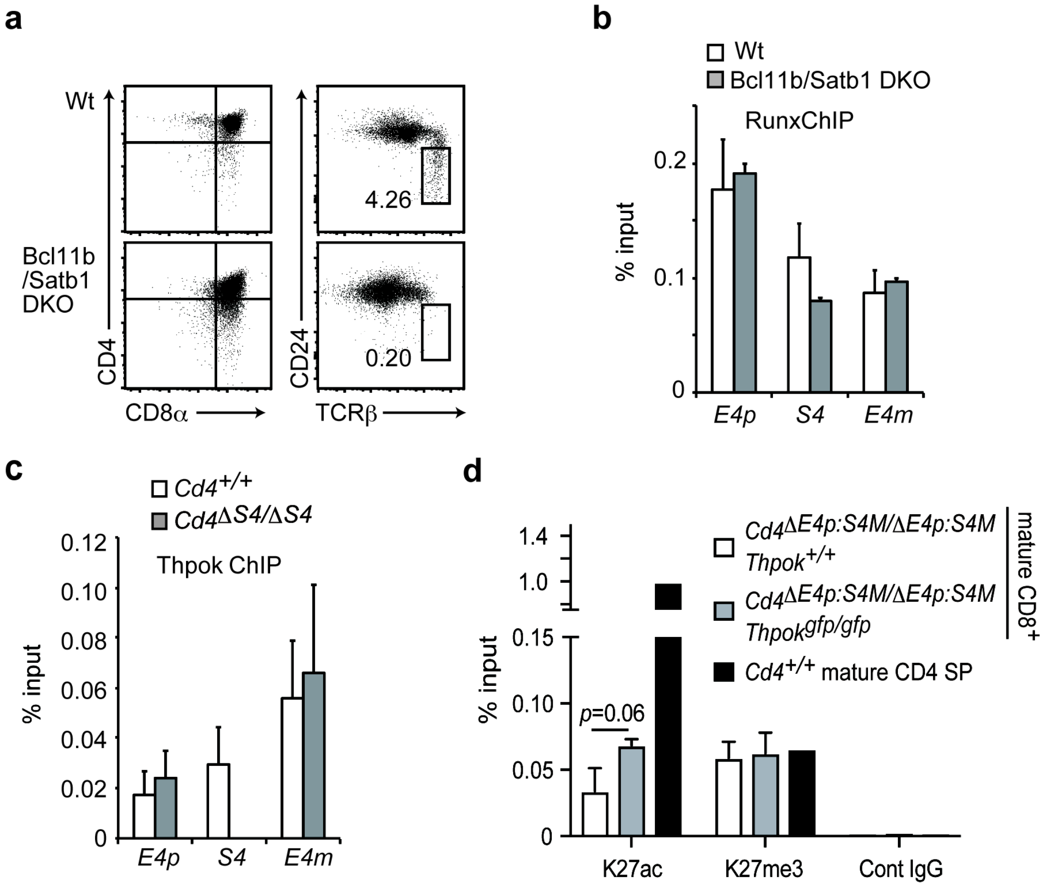

Supplementary Figure 5. Runx/Cbfb and ThPOK binding to E4m enhancer. (**a**) Dot plots showing CD4/CD8 and CD24/TCRβ expression in total thymocytes of wild-type (Wt) and *Bcl11b*$^{F/F}$*: Satb1*$^{F/F}$*: Cd4-Cre* (Bcl11b/Satb1 DKO) mice. Numbers in the indicated region (CD24^{lo}TCRβ^{hi}) indicate respective cell percentages. Development of mature (CD24^{lo}TCRb^{hi}) thymocytes is severely impaired in Bcl11b/Satb1 DKO mice. (**b**) Graph showing summary of three independent ChIP-qPCR experiments for Runx bindings to *Cd4* proximal enhancer (*E4p*), *Cd4* silencer (*S4*), and *Cd4* maturation enhancer (*E4m*) in total thymocytes of wild-type (Wt) and *Bcl11b*$^{F/F}$*: Satb1*$^{F/F}$*: Cd4-Cre* (Bcl11b/Satb1 DKO) mice. Means ± SD. (**c**) Graph showing summary of three independent ChIP-qPCR experiments for ThPOK bindings to *Cd4* proximal enhancer (*E4p*), *Cd4* silencer (*S4*), and *Cd4* maturation enhancer (*E4m*) in peripheral CD4^{+} T cells of Cd4$^{+/+}$ and *Cd4*$^{\Delta S4/\Delta S4}$ mice. Means ± SD. (**d**) Graph showing histone modifications at the *Cd4* maturation enhancer in mature CD8^{+} mature thymocytes. One representative result of two experiments, each of which comprised of three independent ChIP. Means ± SD. Level of histone modifications in control CD4 SP thymocytes are shown as references. *P*-value was obtained by Welch's test.

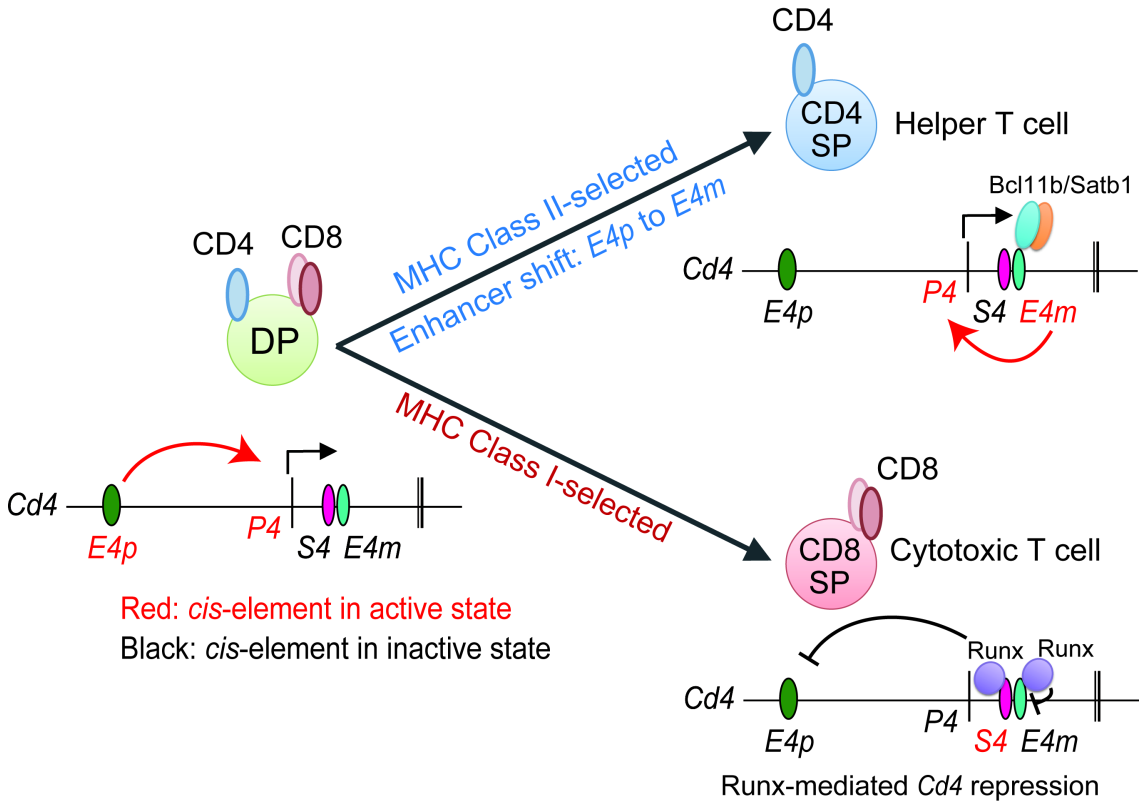

Supplementary Figure 6. Summary diagram of *Cd4* regulation. Activation of *Cd4* proximal enhancer (*E4p*) is critical for the induction of *Cd4* expression during transition from DN to DP stage. During maturation of MHC class II-selected thymocytes, activation of *Cd4* maturation enhancer (*E4m*), at least in part by Bcl11b and Satb1, is essential to establish stably inheritable CD4 expression. Inactivation of *E4p* and *E4m* by Runx complexes in a *S4* silencer-dependent and -independent manner, respectively, establishes silenced state in the *Cd4* locus, erasing CD4 expression in MHC class I-selected thymocytes.

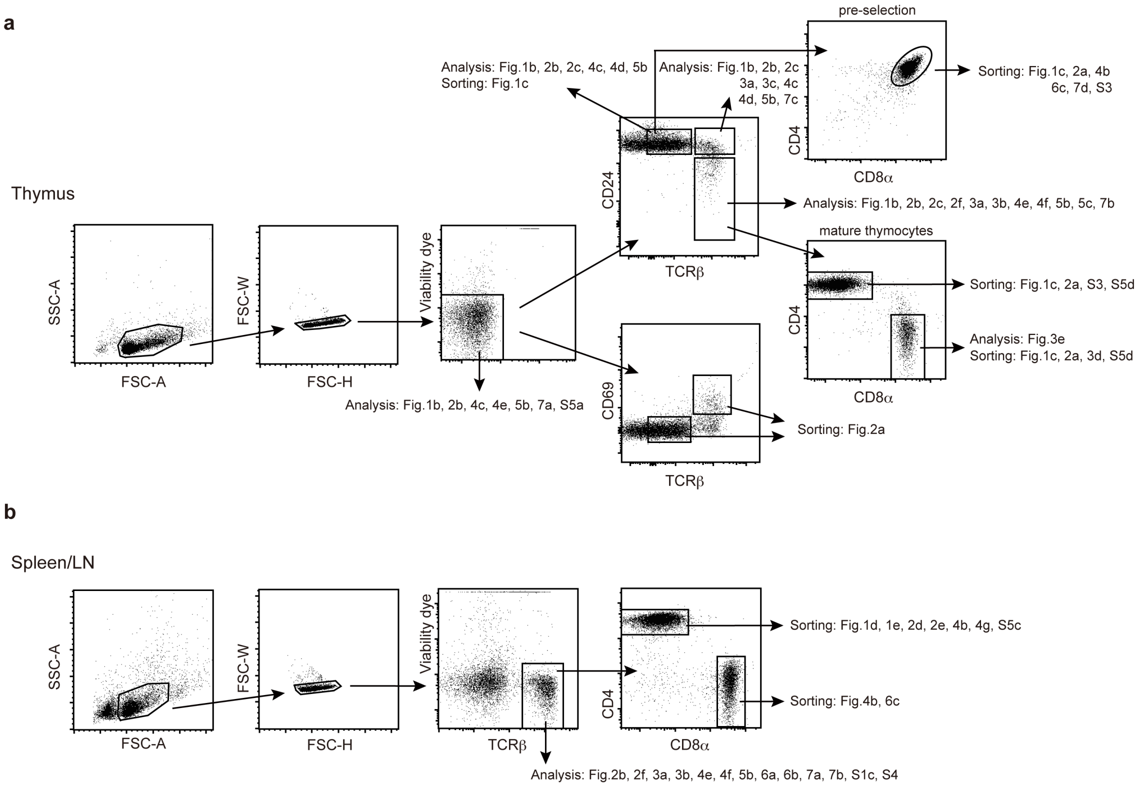

Supplementary Figure 7. Gating strategies used for cell analyses and sorting. Representative FACS plots showing a gating strategy for analyses and cell-sorting of the thymocytes (**a**) and peripheral lymphoid tissues such as spleen and lymph nodes (**b**).

Supplementary Table 1.

Primers for ChIP-qPCR

Target	Forward primer (5'-3')	Reverse primer (5'-3')
Cd4-E4p	TCTCCAAAGGGTAACAGGTGTCAG	TGTGACTTACAAAGGCTGCCTCCA
Cd4-E4m	GGATGCTGCGCTTACTCTTT	CTTCAGGTTTCCGCTGCTAC
Cd4-S4	CCTTGTGTGGTCCCTCTCTTTG	GCAACAACCACCCTTCACAGG
Negative control region	Mouse Negative control primer set 1 (ACTIVE MOTIF: 71011)	
